# Renal cell carcinoma in a patient with staghorn stones: A case report

**DOI:** 10.1016/j.ijscr.2023.108678

**Published:** 2023-08-18

**Authors:** Handaru Satwikananda, Made Adi Wiratama, Karinda Triharyu Caesari Putri, Doddy Moesbadianto Soebadi

**Affiliations:** a Department of Urology, Faculty of Medicine, Universitas Airlangga/Dr. Soetomo General-Academic Hospital, Surabaya, East Java, Indonesia; b Department of Urology, Faculty of Medicine, Jenderal Soedirman University/Prof. Dr. Margono Soekarjo Hospital, Purwokerto, Central Java, Indonesia

**Keywords:** Staghorn stone, Renal stone, Renal cell carcinoma, RCC, Kidney cancer, Radical nephrectomy

## Abstract

**Introduction and importance:**

Staghorn stone fills the renal pelvic and two or more branches of renal calyces. The incidence of staghorn stones is between 10 and 20 % of all urinary tract stones. We report the case of a man with right staghorn stones and renal mass who underwent right radical nephrectomy with pathology anatomy result of renal cell carcinoma (RCC).

**Case presentation:**

A 56-year-old man came with a complaint of right flank pain for two months. Physical examination is within normal limits, but an abdominal CT scan revealed a staghorn stone with enhancing mass in the upper pole of the right kidney. Patient subsequently underwent right radical nephrectomy. Pathology examination revealed RCC.

**Clinical discussion:**

The presence of kidney stones in renal malignancy is rare. Kidney stones can be a risk factor for renal cell malignancy, and renal cell malignancies can cause urinary stasis, making it a risk factor for kidney stones. A study conducted by Nugroho and colleagues concluded that renal and caliceal biopsy should be considered in large and chronic renal stone due to potential experiencing kidney malignancy in patient with renal stone. Therefore, early diagnosis and definitive can be carried out.

**Conclusion:**

Kidney stones and malignancy are rarely found. Renal pelvis, and caliceal wall biopsy should be considered in chronic and large renal stone, especially staghorn stone in patient that did not have any signs of malignancy on CT scan. Treatment in such case is focused on the oncological outcome. Therefore, radical nephrectomy is the treatment of choice.

## Highlights

• Coexistence of renal cell carcinoma (RCC) and staghorn stone is a rare case.
• Biopsy of renal pelvis and calyx walls should be considered for old/chronic and large stones, especially staghorn stone.
• Radical nephrectomy is the treatment of choice for patient with RCC and staghorn stone.

## 1 Introduction

Staghorn stones are stones that fill all renal pelvis and branches of calyxes [1,2]. Although, the term ‘staghorn’ provides a description of stone configuration, it does not specifically describe volume criteria and information about stone composition. Previously, it was widely accepted that staghorn stones make up 10–20 % of all urinary tract stones. However, this prevalence is currently reduced to 4 % in developed countries due to early and effective management of kidney stones [2,3].

There is one meta-analysis study by Cheungpasitporn et al. and one prospective cohort study conducted by Van de pol et al. that investigated the risk factors for RCC in patients with kidney stone. From these two studies, there was increasing risk of RCC in individuals with kidney stones [4,5]. However, kidney stone were associated with an increased risk of RCC in men, but not in women. Two other retrospective cohort studies are not included in the meta-analysis also found an increased risk of kidney cancer in patients with urinary tract stones [6]. This increasing risk is associated with chronic inflammation and infection, which can lead to changes in urothelial cell proliferation. This process can lead to tumor development [5].

The author described a case report of a 56-year-old man who is diagnosed with right staghorn stone and right renal tumor. The patient underwent right radical nephrectomy with RCC pathology anatomical results. The approach and handling of these cases will be discussed briefly on the discussion section. The aim of this report is to describe and analyze a case of staghorn stone with RCC as its complication and its management. This case report was reported according to the SCARE guidelines [7].

## 2 Presentation of case

A 56 years old man came with complaints of right flank pain which has been felt for two months. It has been persistent and getting worse since the last two weeks. There was no history of hematuria and stone expulsion. The patient also did not complain of nausea and vomiting. The patient has a history of hypertension and diabetes mellitus, but does not take medication regularly.

On physical examination, the patient's general condition was fair. Examination of the heart and lung was normal. Abdominal examination was unremarkable. Local examination on the flank region showed slight tenderness on the right side.

Laboratory examination obtained a hemoglobin level of 15.9 g/dL. Urea and serum creatinine level were 25.29 mg/dL and 1.01 mg/dL respectively. On a random blood sugar check, blood sugar level was 201 ng/dL. From the examination of serum electrolytes, normal results were obtained with sodium levels of 139 mmol/L and potassium 3.3 mmol/L. Hematuria was not detected in the dipstick urine analysis; instead, only leukocyturia was identified. The urine cytology was negative for malignant cells.

**Fig. 1:**
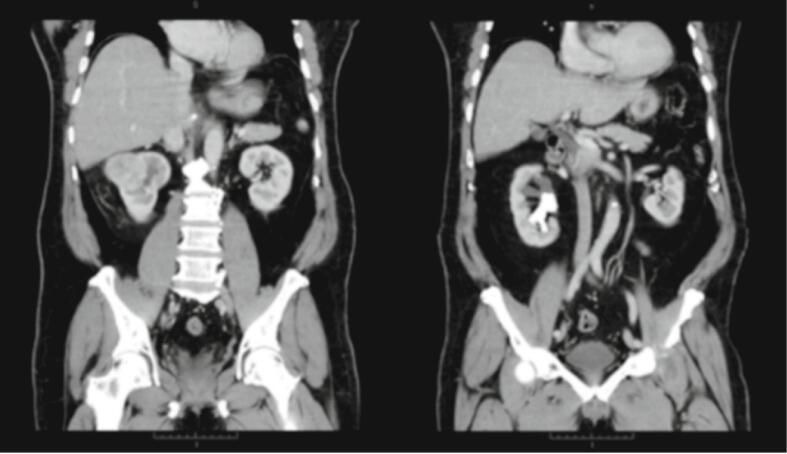
Abdominal CT scan with contrast showing staghorn stones and an inhomogeneous solid mass with contrast enhancement at the upper pole of the right kidney.

On CT scan of the abdomen, a staghorn stone with a size of 4.7 × 4.1 × 1.8 cm was found with an inhomogeneous solid mass in the upper pole of the right kidney with a size of 6 × 5.6 × 4.7 cm with contrast enhancement (Fig. 1). Contralateral kidney, bladder, and the other abdominal organs were within normal limits.

**Fig. 2:**
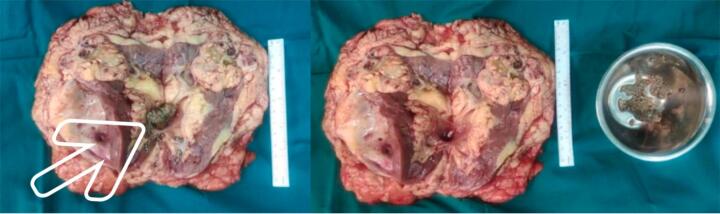
Gross examination of radical nephrectomy showing enlarged kidney sized 18 × 12 × 9 cm and staghorn stone (white arrow) inside the renal pelvic.

**Fig. 3:**
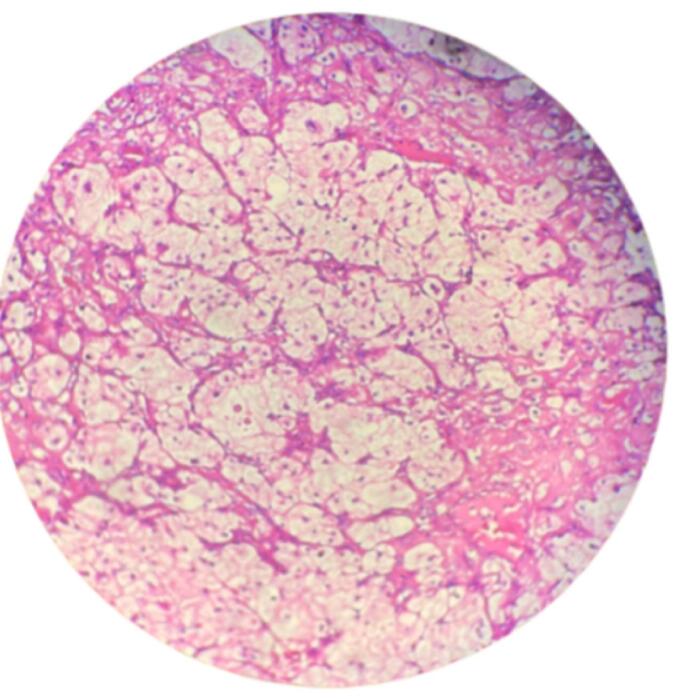
Histologic examination specimen showing the histology of clear cell RCC.

The patient subsequently underwent right radical nephrectomy. Gross examination showed enlarged kidney sized 18 × 12 × 9 cm with solid mass at the upper pole and staghorn stone inside (Fig. 2). No complications were found during the operation. Patient condition was stable after surgery, and was discharged at postoperative day five. Pathology anatomical result showing tumor composed of atypical cells with many cytoplasm, hyperchromatic round oval nucleus, and arranged in solid groups infiltrating the stroma of connective tissue, which corresponds to the description of clear cell RCC (Fig. 3).

## 3 Discussion

Kidney stone is the most common condition in the urinary tract, affecting 12 % of the world's population with annual incidence of 600,000 in America [8]. In male population, the incidence of kidney stones is 2.2 to 3.4 higher than in female, but recent evidence suggests that this gender ratio is changing [9]. Staghorn stone is defined as stones that fill the entire renal pelvis and calyces of the kidney. It was widely accepted that staghorn stone accounted for 10–20 % of all kidney stones, but now it decreased to 4 % in developed countries due to early and effective treatment of kidney stones [3].

Kidney cancer accounts for around 2 % of all cancer diagnoses and cancer deaths worldwide, with generally higher incidence rates in developed countries [10]. The incidence of kidney cancer continues to increase with age, with a peak incidence around the age of 75 years. The incidence of kidney cancer is two times higher in men compared to women. This pattern has been reported repeatedly over time, across countries, and by age group, and so far remains unexplained [11]. Most cases of RCC in developed countries are discovered incidentally on imaging, usually by Ultra Sonography (USG), CT scan, or Magnetic Resonance Imaging (MRI). Only 10 % of kidney cancer patients present with the “classic triad”: hematuria, flank pain, and a palpable mass. Other common symptoms include fever, weight loss and leukocytosis. Approximately 20 % of patients also have paraneoplastic syndromes, including hypercalcemia due to parathyroid hormone-related peptides, polycythemia due to synthesize of erythropoietin, Cushing's syndrome due to adrenocorticotropic hormone (ACTH) and hypertension due to excessive secretion of renin. RCC is rarely suspected in the presence of left-sided varicocele due to left renal vein obstruction [9]. Management of kidney cancer includes surgery in localized-locally-advanced cases, and immunotherapy in metastatic cases.

RCC is the seventh most common histological type of cancer in the Western hemisphere and is increasing in prevalence, representing 1 % to 3 % of all malignant visceral neoplasms. Approximately 40 % of RCC patients die due to disease progression, making it the most lethal malignant urological tumor. Most RCCs are incidentally found on radiological examination, either in patients who come because of urological symptom or not. As many as two-thirds of RCC cases are male [12,13]. RCC is the most common kidney malignancy, accounting for approximately 85 % of cases [14]. In this case report the type of malignancy is renal cell carcinoma, which is the majority of cases of renal malignancy.

Although the incidence of kidney stones has a high prevalence, not many cases have reported kidney cancer and kidney stones that occur synchronously. J. W. Lee et al. reported one case of ipsilateral RCC and Transitional Cell Carcinoma (TCC) in one kidney and kidney stones in the contralateral side. The combination of RCC in one kidney and staghorn stones in the other kidney is very rare [15]. In this case report RCC and staghorn stone was found in right kidney.

Kidney stone is a risk factor for kidney cancer. A meta-analysis study conducted by W. Cheungpasitporn et al., showed that there was a significant association between patients with a history of kidney stone having an increased risk of the two most common types of kidney cancer, namely RCC and TCC, with an overall rate of 1.76 times for RCC and 2.14 times for TCC [4]. The study explained that risk factors for kidney cancer include hypertension, obesity, use of analgesic drugs, renal cyst disease and exposure to the occupation environmental [4,5]. The risk factors found in the patient of this case report are in accordance with the study, where the patient was found to have hypertension and obesity.

The mechanism for the occurrence of RCC and TCC in patients with kidney stones is caused by infection and chronic irritation of the renal mucosa [16]. Literature compiled by Shruti Gupta and colleagues in the journal “Seminar in Cancer Biology” explains that the relationship between kidney stones and malignancy in the kidney especially TCC is mainly caused by chronic inflammation and irritation. Chemokines and cytokines secreted by inflammatory cells leads to proliferative changes in urothelial cells that cause neoplastic cells growth and malignancy [17]. Several studies propose an association between urolithiasis and the onset of RCC; however, the specific mechanism has not been fully understood. RCCs are believed to stem from the renal tubules' proximal convoluted segments [18]. In theory, the formation of RCC might be connected to salts responsible for stone formation found in the filtrate within these proximal tubules. The existence of these solutes might influence cellular metabolism, potentially contributing to the initiation of RCC [5].

CT scan or MRI is used to characterize kidney mass [19]. Abdominal CT scan provides information about the function and morphology of the contralateral kidney, extension of the primary tumor, venous involvement, enlargement of locoregional lymph nodes, and condition of the adrenal glands and other solid organs. CT angiography with abdominal contrast is useful in selected cases where detailed information about the renal vascular supply is needed [20,21]. If CT scan results are inconclusive, contrast enhanced ultrasound (CEUS) is an important alternative for further characterizing renal lesions [22].

Ultrasonography has lower sensitivity and specificity than CT scan for the diagnostic process of kidney stones, but does not emit radiation. However, CT scans often contain incidental findings, which may or may not be clinically important but often point to further analysis or invasive testing. In this case, the findings that were found in addition to kidney stones is a mass in the kidney. Overall, CT scan is highly sensitive and specific for imaging stones in patients with renal colic, which is important for diagnosis and decision-making regarding surgical management due to the superior anatomic detail obtained [23]. In this study, an abdominal CT scan was performed with contrast and obtained staghorn stones with a size of 4.7 × 4.1 × 1.8 cm, classified as grade III based on the Guy's stone score [24] with an inhomogeneous enhancing solid mass in the upper pole of the right kidney with a size of 6 × 5.6 × 4.7 cm.

In cases of chronic urolithiasis, there's a higher risk that it might progress into TCC. The distinction between TCC and RCC lies in their presentation regarding hematuria and positive urine cytology in TCC, while RCC is less commonly associated with these indicators. Moreover, distinguishing between TCC and RCC through imaging is relatively straightforward. TCC typically originates from the pelviocalyceal system, whereas RCC arises in the renal parenchyma [25]. Another difference is that TCC tends to be more endophytic, while RCC is exophytic. Considering the patient's symptoms, who did not experience any hematuria, along with negative urine cytology, and a mass located in the renal parenchyma and exhibiting exophytic growth, we suggested that the patient had RCC.

Open surgery is used to be the mainstay of treatment for kidney stone, but nowadays it has been replaced by minimally invasive procedures such as; Extracorporeal Shockwave Lithotripsy (ESWL), ureteroscopy, and percutaneous nephrolithotomy (PCNL) [26]. There have been studies comparing PCNL and open surgery in the management of staghorn stone, and demonstrated comparable stone clearance, but with less bleeding, shorter operative times, fewer operative complications, and shorter hospital stays for PCNL [27].

The management of RCC is a challenge in itself. An effective multidisciplinary approach to the process of diagnosis, staging, and treatment. Guidelines recommend active surveillance, thermal ablation, partial nephrectomy, radical nephrectomy, cytoreductive nephrectomy and immunotherapy as various modalities for different stages of RCC. However, open radical nephrectomy is most widely adopted as an option for treatment at various stages of disease because of its cost-effectiveness, applicability at various stages, and reduced follow-up costs [28]. While it is certainly possible to perform a partial nephrectomy in a T1b patient, the complexity of this tumor, as indicated by its R.E.N.A.L nephrometry score (score 10), raises the potential for peri-operative complications [29]. Given that the patient's renal function is still favorable, we have chosen to proceed with a radical nephrectomy.

In a study conducted by Nugroho and colleagues, it was concluded that patients with kidney stones have increasing risk to suffer kidney malignancy later. It also said that patients with staghorn stones and complex/large stones with a size >20 mm are associated with malignancy in the renal pelvis. Biopsy of the renal pelvis and calyx walls in cases of old/chronic stones and large stones, especially staghorn stones should be considered. This is due to the poor prognosis in progressive squamous cell carcinoma (SCC), so that early diagnosis and definitive treatment can be carried out [30]. Biopsy of the renal pelvis and calyx walls is suggested for patients who have old/chronic and large stones without any signs of malignancy on CT scan. The biopsy can help detect cancer early in patients with large kidney stones, especially staghorn ones. If the CT scan shows a tumor especially the enhancing mass that suggestive to malignant, then a biopsy is not necessary.

Postoperative surveillance allows the urologist to monitor postoperative complications, renal function, local recurrence, recurrence in the contralateral kidney and development of metastases. Renal function and postoperative complications are usually assessed by history taking, physical examination, and measurements of serum creatinine and hemoglobin at four to six weeks postoperatively. Long-term monitoring of serum creatinine is recommended especially in patients with impaired renal function before surgery or a significant increase in creatinine after surgery. Early diagnosis of local and contralateral renal recurrence (<2 % incidence) is useful because the most effective treatment is surgical resection (grade 3) [31]. Recurrence can be associated with positive, multifocal and grade surgical margins [32].

## 4 Conclusion

Kidney stone and kidney malignancy are rarely found together. Kidney stone can be a risk factor for kidney malignancy and kidney malignancy can cause urine stasis so it will become risk factor for kidney stone. Biopsy of the renal pelvis and calyx wall should be considered in case of old/chronic and large stones, especially staghorn stone, that did not have any signs of malignancy on CT scan. It is intended for early diagnosis and appropriate definitive management of the patient. The treatment of these two conditions that found together focuses on the oncological outcome. In this case the authors report a 56-year-old male patient with RCC and staghorn stones in the right kidney. The patient underwent a right radical nephrectomy procedure and obtained pathological results of renal cell carcinoma anatomy.

## Informed consent

Written informed consent was obtained from the patient for publication and any accompanying images. A copy of the written consent is available for review by the Editor-in-Chief of this journal on request.

## Funding

This research did not receive any specific grant from funding agencies in the public, commercial, or not-for-profit sectors.

## Ethical approval

There was no requirement for ethical approval in order to administer treatment or conduct an investigation on this particular patient. Ethical clearance is not required because the surgical treatment in this patient is the gold standard for kidney malignancy. This case later used as case report after the histo-pathology results shown as clear cell renal cell carcinoma (RCC), which is a rare entity case that concomitant with staghorn stone.

## CRediT authorship contribution statement

• Handaru Satwikananda: Conceptualization, Design, Resources, Data Collection, Writing Manuscript
• Made Adi Wiratama: Conceptualization, Design, Review and Editing
• Karinda Triharyu Caesari Putri: Investigation, Resources, Supervision, Critical Review
• Doddy Moesbadianto Soebadi: Conceptualization, Resources, Supervision, Review and Editing

## Registration of research studies

Not applicable.

## Guarantor

Doddy Moesbadianto Soebadi.

## Declaration of competing interest

The authors declare that there are no conflicts of interest.
